# Advanced Necklace for Real-Time PPG Monitoring in Drivers

**DOI:** 10.3390/s24185908

**Published:** 2024-09-12

**Authors:** Anna Lo Grasso, Pamela Zontone, Roberto Rinaldo, Antonio Affanni

**Affiliations:** 1 Polytechnic Department of Engineering and Architecture, University of Udine, 33100 Udine, Italy; anna.lograsso@uniud.it (A.L.G.); antonio.affanni@uniud.it (A.A.); 2 Department of Electrical, Electronic, Telecommunications Engineering and Naval Architecture, University of Genoa, 16145 Genoa, Italy; pamela.zontone@unige.it

**Keywords:** photoplethysmography, heart rate monitoring, electrocardiogram, motion artifacts, sliding DFT

## Abstract

Monitoring heart rate (HR) through photoplethysmography (PPG) signals is a challenging task due to the complexities involved, even during routine daily activities. These signals can indeed be heavily contaminated by significant motion artifacts resulting from the subjects’ movements, which can lead to inaccurate heart rate estimations. In this paper, our objective is to present an innovative necklace sensor that employs low-computational-cost algorithms for heart rate estimation in individuals performing non-abrupt movements, specifically drivers. Our solution facilitates the acquisition of signals with limited motion artifacts and provides acceptable heart rate estimations at a low computational cost. More specifically, we propose a wearable sensor necklace for assessing a driver’s well-being by providing information about the driver’s physiological condition and potential stress indicators through HR data. This innovative necklace enables real-time HR monitoring within a sleek and ergonomic design, facilitating seamless and continuous data gathering while driving. Prioritizing user comfort, the necklace’s design ensures ease of wear, allowing for extended use without disrupting driving activities. The collected physiological data can be transmitted wirelessly to a mobile application for instant analysis and visualization. To evaluate the sensor’s performance, two algorithms for estimating the HR from PPG signals are implemented in a microcontroller: a modified version of the mountaineer’s algorithm and a sliding discrete Fourier transform. The goal of these algorithms is to detect meaningful peaks corresponding to each heartbeat by using signal processing techniques to remove noise and motion artifacts. The developed design is validated through experiments conducted in a simulated driving environment in our lab, during which drivers wore the sensor necklace. These experiments demonstrate the reliability of the wearable sensor necklace in capturing dynamic changes in HR levels associated with driving-induced stress. The algorithms integrated into the sensor are optimized for low computational cost and effectively remove motion artifacts that occur when users move their heads.

## 1. Introduction

The analysis of physiological signals has been gaining importance in various research areas, contributing to enhancing quality of life and evaluating public health. Among these signals, heart rate variability (HRV) is linked to the autonomic nervous system of a subject, so it serves as a valuable measure for assessing the individual’s cardiac health, stress level, and overall well-being [1]. The development of wearable devices for heart rate monitoring through photoplethysmographic (PPG) signals has revolutionized the way we track and manage health [2], particularly by monitoring stress levels of drivers and during daily activities [3,4]. A PPG signal primarily measures the variations in blood volume within the tissues [5]. When the heart pumps blood, the volume of blood in the arteries and capillaries fluctuates with each heartbeat. The PPG sensor detects these changes, providing a waveform that correlates with the cardiac cycle. PPG-based heart rate monitoring offers a non-invasive, continuous, and convenient method to track physiological responses to stress and physical effort in real time [6,7]. For drivers, maintaining a calm and focused state is essential for road safety [8,9,10]. Stress can adversely affect reaction times and decision-making abilities, increasing the risk of accidents [11,12]. By monitoring heart rate variability, it is possible to detect early signs of stress and implement timely interventions to enhance the driver’s well-being and safety [13,14,15]. In daily life, especially during physical activities, heart rate monitoring is invaluable for maintaining optimal health and fitness. It helps individuals gauge exercise intensity, ensuring they stay within safe limits to avoid overexertion and reduce the risk of injuries. Additionally, regular monitoring can provide insights into cardiovascular health, allowing early detection of potential issues and enabling proactive health management.

A PPG sensor uses a light source, typically a light-emitting diode (LED), and a photodetector. The light is emitted into the skin, and the photodetector measures the amount of light either absorbed or reflected by the blood in the tissue. The amount of absorbed light increases with the blood volume in the capillaries and decreases when the volume is lower [16]. By analyzing the periodicity of the PPG signal, the heart rate can be determined. The largest peaks in the PPG waveform correspond to the systolic phase of the cardiac cycle, during which the heart pumps blood into the arteries. Counting these peaks over time provides an accurate measure of the heart rate. Unfortunately, the use of PPG sensors is prone to motion artifacts that introduce noise and distortion into the signal. Movement alters the sensor’s contact with the skin (especially in wrist sensors), causing fluctuations unrelated to blood flow and making it difficult to isolate the actual physiological data. Motion can also create extraneous peaks and troughs in the logged signal, leading to incorrect measurements of heart rate and other parameters [7].

The primary focus of our research is to monitor the responses of drivers, who typically exhibit limited movements that mainly involve arm and leg motions for steering and pedal operations. To address these issues, this paper presents the development of an innovative wearable device in the form of a necklace that is designed to measure PPG. Using this kind of sensor offers several advantages over traditional PPG sensors. Firstly, the necklace design ensures consistent contact with the skin, reducing motion artifacts that commonly affect wrist- or finger-based sensors. This improves the accuracy and reliability of measurements. Secondly, a necklace is more comfortable for continuous use, which makes it ideal for long-term monitoring. Furthermore, to analyze the sensor’s performance, two algorithms for HR estimation are tested, with the aim of removing or attenuating motion artifacts in PPG signals: a modified version of the mountaineer’s method (MM) [17], which incorporates a new approach for peak recognition compared to its previous implementation, and a sliding discrete Fourier transform (SDFT) [18,19]. The latter, by converting the signal from the time domain to the frequency domain, effectively isolates the heart rate component from other physiological and noise signals. Additionally, the sliding window approach enables the algorithm to adapt to signal variations, maintaining accurate HR estimation even under varying conditions. The efficiency and accuracy of the two algorithms are evaluated using data obtained from the necklace sensor through experiments conducted in a simulated driving scenario and on a public dataset with recordings of people carrying out physical exercises or daily life activities [20,21,22]. Evaluation rules and metrics are defined that facilitate a comparison between the different approaches. The obtained results show a satisfactory level of accuracy for all the tested subjects, demonstrating the reliability of the SDFT for real-time heart rate estimation from PPG signals. Regarding the mountaineer’s algorithm, we verify that it works well on data collected using the necklace sensor but has some limitations when tested on signals heavily affected by motion artifacts, such as those available in public datasets recorded using a wrist-worn PPG sensor. However, we compare the results obtained using a public dataset with those reported in the literature. The chosen dataset currently aligns best with our objective, as it involves subjects walking or running on a treadmill. This allows us to verify that, despite these data being quite different from those obtained with our necklace sensor and being more prone to motion artifacts, the results achieved through the implementation of our algorithms are sufficiently satisfactory.

In summary, the main contributions of this paper are as follows.

- We develop an innovative necklace sensor that enables real-time monitoring of human vital signs.
- We are able to monitor drivers’ responses, as drivers typically exhibit limited movements, by recording high-quality PPG signals with minimal interference.
- We implement systems based on a low-power design and simplified low-computational-cost algorithms that enable real-time heart rate estimation.
- We create an enhanced version of the mountaineer’s algorithm, which is already present in the literature, making it suitable for integration into our novel sensor.

The paper is organized as follows. Section 2 describes the novel necklace sensor we use in our experiments and its PCB design. Section 3 outlines the characteristics of the PPG signal and its relationship with human vital signs, particularly with the cardiac cycle. In Section 4, we present the two computationally efficient algorithms implemented in the microprocessor to extract the heart rate from the PPG signal, which are test in terms of performance for the developed system. In Section 5, we first examine the experimental results obtained using the necklace sensor during preliminary desk tests, followed by the results obtained during driving simulator tests carried out in our BioSensLab laboratory. This section also shows some results using a public dataset. In Section 6, we discuss the experimental results. Finally, some conclusions are drawn in Section 7.

## 2. Sensor Description and PCB Design

**Figure 1:**
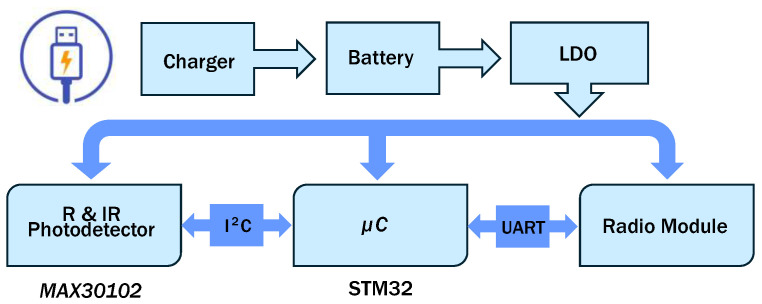
Block diagram of the developed sensor.

This section presents the designed wearable necklace sensor that was briefly introduced in [23]. The developed sensor’s block diagram, shown in Figure 1, features the MAX30102 sensing element from Maxim Integrated (San Jose, CA, USA), which uses the I^2^C protocol for configuring red (R) and infrared (IR) LED power and duty cycles. It acquires the photodetector signal, which is digitized at up to 18-bit precision using a sigma–delta ADC.

The sensor is controlled by an STM32G0B1KET6 microcontroller from ST Microelectronics (Plan-les-Ouates, Geneva, Switzerland) operating at a 24 MHz clock frequency and communicating with the sensor via I^2^C at 100 kHz. The sensor is designed to sample at 100 Hz (every 10 ms) with 18-bit resolution for both the R and IR channels. The LEDs have a 411 μs pulse width (4% duty cycle) and a 12 mA driving current. The microcontroller detects PPG signal peaks to extract HR and SpO_2_ data, as will be detailed below.

Data are transmitted to a low-power WiFi module (USR-C216) via UART at 115.2 kbps, allowing users to access data on a laptop or smartphone and to choose between raw data or processed HR and SpO_2_ values. The sensor runs on a 3.7 V, 500 mAh LiPo battery, with a dual LDO regulator providing 3.3 V for the microcontroller and WiFi module and 1.8 V for the MAX30102. The battery is rechargeable via a micro-USB connector and an internal charger set to 300 mA. As already mentioned, the current sensor prototype is equipped with a WiFi module. However, in view of a future low-power implementation, this could be replaced with any other alternative transmission module (e.g., Bluetooth Low Energy), as shown in Figure 1.

**Figure 2:**
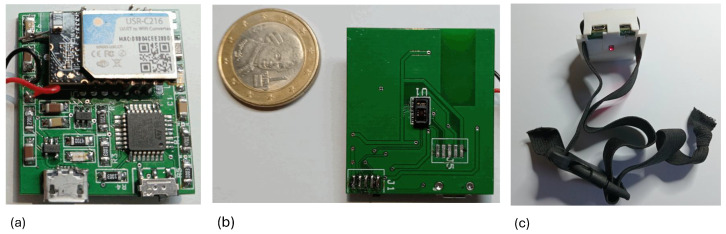
PCB realization of the sensor: (**a**) top layer and (**b**) bottom layer. The sensing element, which comes into contact with the skin, is located exclusively on the bottom layer. (**c**) The necklace in its 3D printed case. The elastic band can be adjusted using a buckle.

The sensor, built on a two-layer PCB measuring 32 × 36 mm^2^, is shown in Figure 2a,b. The bottom layer contains only the sensing element and ensures contact with the skin by exposing the sensing area outside of the plastic case. For prototyping, we used SMD 1206 (Surface Mount Device, chip package type 1206) passive components, but production dimensions can be reduced with smaller packages.

The system consumes 85 mA (0.28 W) during WiFi transmission of the raw data stream, allowing for 6 h of continuous operation without recharging. When transmitting only alarms for abnormal conditions, it consumes 8 mA (28 mW), enabling 2.6 days of operation without recharging. Additionally, we designed and 3D-printed a housing for the sensor, which is worn around the neck with an adjustable elastic band. Figure 2c shows the necklace, whereas in Section 5.2, a volunteer wearing the sensor necklace during the experiments conducted in a simulated driving scenario will be shown.

## 3. PPG Signal Properties

**Figure 3:**
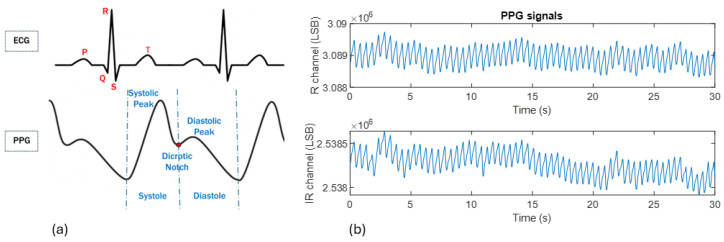
(**a**) Example of PPG and ECG waveform signals and their characteristic parameters; (**b**) example of PPG signals acquired by the necklace sensor from the R and IR channels.

The extraction of the PPG signal is a sophisticated process that leverages optical techniques to provide valuable insights into cardiovascular health. It involves the interaction of light with human tissues. Variations in light intensity correspond to changes in blood volume within the tissue that are driven by the cardiac cycle [24]. As shown in Figure 3a, the PPG waveform consists of several distinct features. The systolic peak, which is the highest point in the waveform, occurs during the systolic phase of the cardiac cycle, when the heart pumps blood, causing an increase in blood volume and light absorption in the tissue. The dicrotic notch, representing a small dip that follows the systolic peak, reflects the transient drop in blood volume as the aortic valve closes. The diastolic peak, which is a secondary peak or wave following the dicrotic notch, represents the diastolic phase when the heart relaxes and fills with blood.

Figure 3b shows PPG signals acquired by the necklace sensor from the R and IR channels. As we can see, the PPG signal exhibits a distinct periodicity reflecting the cyclical nature of the cardiovascular system. This periodicity is characterized by rhythmic oscillations in the signal, which are typically synchronized with the cardiac cycle. Nevertheless, it is generally difficult to discern the sequence of systolic and diastolic peaks in the recorded PPG signals.

In particular, PPG and ECG signals are interrelated components of the cardiovascular monitoring ecosystem, with PPG reflecting the blood volume changes resulting from the heart’s electrical activity as captured by the ECG. Specifically, the systolic peak of the PPG signal occurs shortly after the R-wave of the ECG signal. This time delay is known as the pulse transit time (PTT) and represents the time it takes for the blood pressure pulse to travel from the heart to the peripheral site where the PPG sensor is located.

In addition to its crucial application for heart rate estimation, the PPG signal can be used for measuring oxygen saturation (SpO_2_), which is a critical parameter for assessing respiratory and cardiovascular health. SpO_2_ measurement is based on detecting peaks in PPG signals in order to analyze variations caused by different blood oxygenation levels. In detail, to determine SpO_2_, one can identify the local minimum occurring within one second before each validated peak and then apply the following equation [25]:

(1) $${\mathrm{SpO}}_{2}=110-25\frac{R_{AC}/R_{DC}}{IR_{AC}/IR_{DC}}.$$

Here, $R_{AC}$ and $IR_{AC}$ denote the differences between each peak value and its preceding local minimum in the R and IR channels, respectively. Similarly, $R_{DC}$ and $IR_{DC}$ represent the values of the local minima before each peak in the R and IR channels, respectively.

## 4. Methodology

Implementing efficient algorithms for estimating the heart rate from a PPG signal is necessary to ensure real-time, accurate monitoring, particularly in wearable devices with limited computational power. Efficient algorithms help maintain reliable HR detection despite noise and motion artifacts.

**Figure 4:**
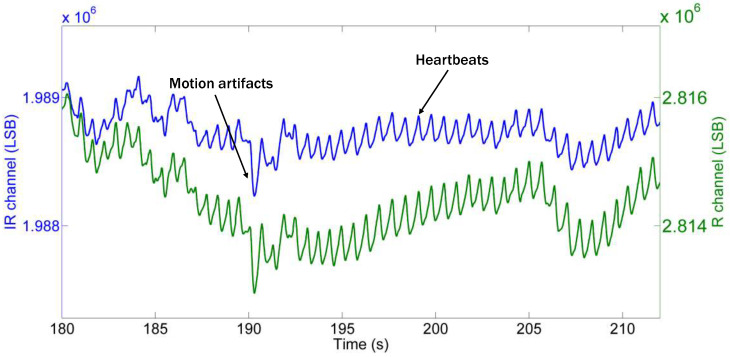
Raw data acquired from the R and IR channels. The peaks corresponding to heartbeats have a much smaller amplitude compared to the overall signal amplitude, as do the baseline and motion artifacts.

PPG devices are sensitive to light absorption and reflection changes affected by external factors. They detect any blood volume or tissue composition changes, which lead to signal contamination. Variations in skin, ambient light, temperature, and anatomy, along with sensor attachment and contact stability, can cause signal inconsistencies. For these reasons, the collected PPG signals (see Figure 4), which represents the R and IR data obtained using the necklace sensor, exhibit various problems, such as small PPG peaks, baseline wandering, and motion artifacts. Small PPG peaks are typically caused by noise, low signal amplitudes, or poor sensor contact with the skin. They can obscure the true pulsatile signal. Furthermore, changes in the DC component of the PPG signal, which is the baseline level around which the pulsatile (AC) component varies, can be due to various factors such as changes in skin tone, the environmental temperature, or changes in blood volume due to changes in posture. Baseline deviations make it challenging to isolate the AC component, which is essential for accurate calculation of the pulse and SpO_2_. Finally, the movement of the sensor or of the subjects themselves can introduce significant noise into the PPG signal. This includes voluntary movements (like walking or talking) and involuntary movements (like shivering). Motion artifacts can also mimic or obscure the true pulsatile signal, making it difficult to distinguish between actual physiological changes and artifacts. These issues hinder the accurate detection of heartbeats and the calculation of reliable physiological parameters such as heart rate and oxygen saturation, thus leading to incorrect estimates and reduced data reliability.

As can be seen in Figure 4, the heartbeats exhibit amplitudes of a few hundred least-significant bits (LSBs), while the baseline measures around one million LSBs. Note that 1 LSB corresponds to a quantization step equal to 15.63 pA. The sensor provides a digital output in I^2^C format, so for convenience, we refer to the raw data transmitted by the sensor. The baseline wandering caused by the user’s slow movements can range from a few hundred LSBs to around several thousand LSBs. Additionally, sudden neck movements produce spikes in the signal, which are also in the thousands of LSBs and are thus significantly larger than the heartbeat amplitudes. These artifacts should not be confused with heartbeats by the detection algorithm. Therefore, it is crucial to accurately detect peak instances while robustly removing artifacts. Furthermore, the algorithm needs to be efficient enough to recognize peaks within 10 ms (which corresponds to the sampling interval) while considering the limited computational power and clock frequency of the microcontroller.

### 4.1. Modified Mountaineer’s Method

One of the algorithms used for heart rate estimation is based on the “mountaineer’s method” [17]. Firstly, PPG signals undergo band-pass filtering in the range of [0.5, 10] Hz. This is achieved using a fourth-order Butterworth IIR filter. The peak detection algorithm incorporates a novel approach for peak recognition.

**Figure 5:**
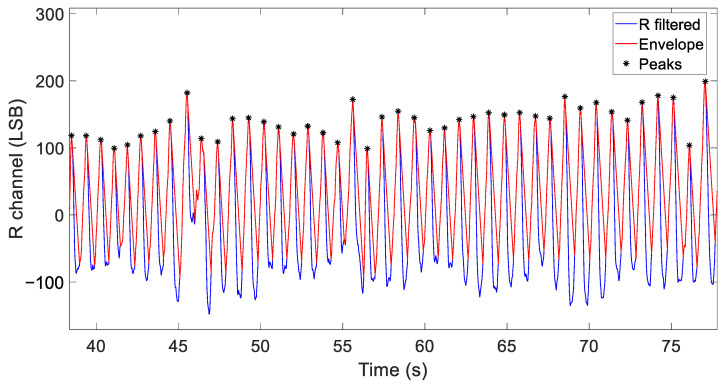
Processed data for peak detection from the R channel—blue line: band-passed R data; red line: envelope detector; black markers: detected peaks.

More specifically, we introduce a synthesized envelope detector designed to track the filtered signal during its rising phase and making it decay linearly during its falling phase, as shown by the red line in Figure 5. The blue line in Figure 5 represents, instead, a detail of the processed PPG signal from the R channel from a single experiment conducted in our lab consisting of an individual wearing the sensor necklace and working while seated at a desk. Potential peaks are identified when the envelope detector shifts its state from a consistent tracking phase (defined as N=5 consecutive samples, or 50 ms) to a consistent decay phase (i.e., 50 ms).

**Figure 6:**
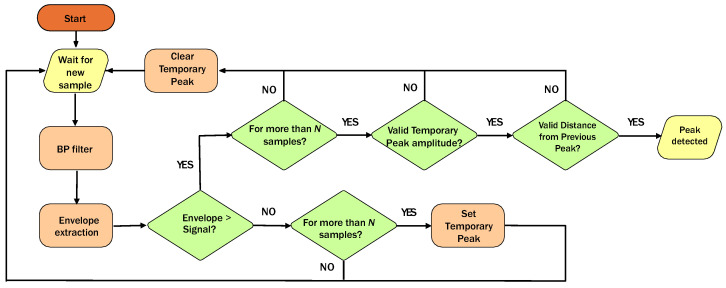
Flowchart of the algorithm implemented to extract the heart rate.

Once detected, these potential peaks and their corresponding time instants are compared to a time-varying hysteresis threshold, which is adjusted based on the magnitude of the last three detected peaks. Additionally, the distances between the instants of consecutive peaks are checked to ensure they are in the range of [350, 2000] ms. A potential peak is confirmed as a valid peak if it passes both the amplitude and distance checks. In Figure 5, valid peak detections are indicated by black asterisks. The flowchart of the implemented algorithm is depicted in Figure 6.

Finally, to eliminate any residual false detections, a fifth-order median filter is applied to smooth the signal. Section 5 discusses the algorithm’s performance under conditions wherein the subject is moving and artifacts are present in the signals. This algorithm can be also used for the estimation of SpO_2_ using Equation (1) in Section 3.

### 4.2. Sliding DFT

Another approach for calculating HRs from PPG signals is through frequency analysis. As shown in Figure 3, the signal is, in fact, normally quasi-periodic, with a frequency corresponding to a normal resting human heartbeat. In the frequency domain, these characteristics are evident in the peaks at the fundamental frequency and its harmonics of the Fourier transform of a signal analyzed in a suitable analysis window. This approach forms the basis of the procedures adopted in [4].

To estimate HRs from PPG signals, we employed the sliding discrete Fourier transform (SDFT) [18,19], which allows for an almost time-continuous analysis in the frequency domain. First, the acquired PPG signals were pre-processed to remove artifacts and noise through a fourth-order Butterworth band-pass filter in the band [0.5 10] Hz. Afterwards, the SDFT was applied to successively overlapping windows of the PPG signal to obtain continuous updating of the frequency spectrum, thus providing real-time estimates of the heart rate.

Assuming consecutive signal blocks of *N* samples, i.e., $x_{i-1}\left(0\right),\ldots ,x_{i-1}\left(N-1\right)$ and $x_{i}\left(0\right),\ldots ,x_{i}\left(N-1\right)$, respectively, we have $x_{i-1}\left(h+1\right)=x_{i}\left(h\right)$, h=0,…,N−2 because of overlapping. The *M*-point DFTs of blocks i−1 and *i* are related by the following expressions:

(2) $$\begin{array}{cc} X_{i-1}\left(k\right) & =\sum_{h=0}^{N-1}x_{i-1}\left(h\right)W^{hk}\\ \; & =x_{i-1}\left(0\right)+W^{k}\sum_{h=0}^{N-2}x_{i-1}\left(h+1\right)W^{hk}, \end{array}$$

(3) $$\begin{array}{cc} X_{i}\left(k\right) & =\sum_{h=0}^{N-1}x_{i}\left(h\right)W^{hk}\\ \; & =\sum_{h=0}^{N-2}x_{i-1}\left(h+1\right)W^{hk}+x_{i}\left(N-1\right)W^{(N-1)k}, \end{array}$$

for k=0,…,M−1, where $W=e^{-j2\pi /M}$. We can choose M>N to increase the frequency resolution to $F_{s}/M$ Hz, corresponding to $F_{s}/M\cdot 60$ beats/s. By combining Equations (2) and (3), we obtain

(4) $$X_{i}\left(k\right)=X_{i-1}\left(k\right)W^{-k}-x_{i-1}\left(0\right)W^{-k}+x_{i}\left(N-1\right)W^{(N-1)k}.$$

Equation (4) allows the DFT values to be calculated recursively, requiring only three complex products for each value of *k*. In addition, depending on the application, it is sufficient to calculate the DFTs for a small number of indices *k*, which saves computation even compared to a fast algorithm such as the fast Fourier transform (FFT). The recursion can, of course, be initiated by setting $x_{-1}\left(h\right)=0$, h=0,…,N−1, and therefore, $X_{-1}\left(k\right)=0$ for all values *k* of interest.

Technically speaking, Equation (4) corresponds for each *k* to a first-order IIR filter:

(5) $$X_{i}=\alpha X_{i-1}+s_{i},$$

where for ease of notation, we drop index *k* and denote $W^{-k}$ as α and $-x_{i-1}\left(0\right)W^{-k}+x_{i}\left(N-1\right)W^{(N-1)k}$ as $s_{i}$. Note that the filter is marginally stable since it has a pole α with a unit module. Keeping in mind a fixed-point implementation, this may lead to undesirable and potentially destructive amplification of the rounding error due to limited precision. Indeed, the recursive Equation (5) can be unfolded to

(6) $$X_{i}=\sum_{k=0}^{i}\alpha^{k}s_{i-k},\;i=0,1,\ldots .$$

Following [26], the effect of the rounding error can be modeled as additive noise $n_{k}$ in the accumulator, leading to a cumulative error for $X_{i}$ computed as in (6) by

(7) $$e_{i}=\sum_{k=0}^{i}\alpha^{k}n_{i-k},\;i=0,1,\ldots .$$

Considering a fixed point implementation with *m* bits, we may assume $|n_{i-k}|\leq \sqrt{2}\cdot 2^{-m}$. (The exact bound depends on the specific processor architecture. Here, we suppose that the computation of the real part contributes the sum of two rounding errors. The same applies for the imaginary part computation.) It is also reasonable to model $n_{i-k}$ as independent random variables with independent real and imaginary parts with zero means and variances $\sigma_{n}^{2}=2\cdot 2^{-2m}/12,$ so that $e_{i}$ can be considered approximately complex Gaussian. Within these hypotheses, by using (7), we can write

(8) $$\begin{array}{c} |e_{i}|\leq \sqrt{2}\cdot 2^{-m}\left(i+1\right) \end{array}$$

(9) $$\begin{array}{c} \sigma_{e_{i}}^{2}=E[|e_{i}{|}^{2}]=2\sigma_{n}^{2}\left(i+1\right). \end{array}$$

As an example, considering a sample frequency $F_{s}=100$ Hz (or $F_{s}=20$ Hz, as another example) and m=32-bit precision, running the algorithm for 24 h will result in approximately $|e_{i}|<3\cdot 10^{-3}$ (or $|e_{i}|<6\cdot 10^{-4}$ at $F_{s}=20$ Hz). As an explicative case, consider a sinusoidal input signal with frequency $f_{0}≃1$ Hz (mimicking a toy PPG signal) and an analysis window of 10 s, so that $N=10F_{s}.$ The corresponding DFT exhibits a peak at $f_{0}$ with an amplitude of N/2, making the effect of the rounding error totally negligible in practice. This is confirmed in our experiments with real PPG signals. The analysis of the spectrum generated by the SDFT allows us to identify the peak frequency corresponding to the heart rate.

## 5. Experimental Results

To test the performance of our measurement system, we implemented on the microprocessor the two algorithms for HR estimation described in the previous section. Here, we show some experimental results obtained considering three different datasets. The first two datasets were obtained by recording the PPG signals from individuals wearing both the necklace sensor (introduced in Section 2) and the ECG sensor described in [27,28]. This allows us to compare the extracted heart rate with a ground truth reference. In the first dataset, an individual sat at a desk and worked on a computer. In the second dataset, subjects drove the dynamic driving simulator available in our BioSensLab [29] at the University of Udine. The algorithms were also tested on a third dataset that was made publicly available for the IEEE Signal Processing Cup 2015 [20]. In this case, subjects walked or ran on a treadmill.

Various metrics that were also used in other studies [4,30] were calculated to evaluate the accuracy performance and reliability of our algorithms applied to each dataset. In particular, we defined $f_{est}\left(i\right)$ and $f_{true}\left(i\right)$ as the *i*-th estimated HR value and the *i*-th true HR value in BPM, respectively (i.e., they represent the HR values in the *i*-th time window for the sliding DFT), and we defined $AE_{i}=\left|f_{est}\left(i\right)-f_{true}\left(i\right)\right|$ as the absolute error used to estimate the accuracy of each HR estimation. We used these three metrics: average absolute error (*av*AE), standard deviation of the absolute error (*sd*AE), and average relative error (*av*RE). These are computed as follows:

(10) $$\begin{array}{cc} \mathrm{av}AE & =\frac{1}{N}\sum_{i=1}^{N}AE_{i} \end{array}$$

(11) $$\begin{array}{cc} \mathrm{sd}AE & =\sqrt{\frac{1}{N}\sum_{i=1}^{N}{\left(AE_{i}-\mathrm{av}AE\right)}^{2}} \end{array}$$

(12) $$\begin{array}{cc} \mathrm{av}RE & =\frac{1}{N}\sum_{i=1}^{N}\frac{AE_{i}}{f_{true}\left(i\right)} \end{array}$$

where *N* is the total number of estimates.

To integrate both algorithms on the microcontroller, we had to convert all values from floating-point to 32-bit fixed-point arithmetic. This led us to evaluate the processing times of both algorithms. More specifically, under worst-case conditions, the mountaineer’s method takes 240 μs to identify a peak, whereas the sliding DFT takes 1.1 ms. Considering that the sampling interval is 10 ms, we confirmed that both algorithms have a computational complexity that is much less than the sampling interval, thus fitting the requirements of our implementation. This will also enable us in the future to implement both algorithms in a cascading manner, allowing one to validate the other.

### 5.1. Preliminary Tests

During some preliminary tests, signals were recorded for a 47-year-old male volunteer sitting at a desk working on a computer and occasionally moving his head. He wore the necklace sensor to acquire the PPG signal (see Figure 2) and a sensor on his chest to measure the ECG signal. During these preliminary tests, we collected 10 tracks, each lasting 120 s, recorded on 10 different days.

The movement of the head introduced noise and motion artifacts on the acquired data, and this allowed us to evaluate the performance of the heart rate estimation algorithms and their capability to remove artifacts caused by head motion. Indeed, to determine whether the extracted heart rate was influenced by artifacts, we used the Pan–Tompkins algorithm [31] to extract the heart rate from the ECG signal. This was used as a reference to assess the reliability of the estimated heart rate. We then verified the performance of the two algorithms in terms of heart rate calculation by comparing the obtained estimates with the reference value extracted from the ECG.

Table 1 lists the metric values of the two proposed algorithms (MM and SDFT) for all 10 traces. It also summarizes the performance for the set of recordings by showing the average values.

**Figure 7:**
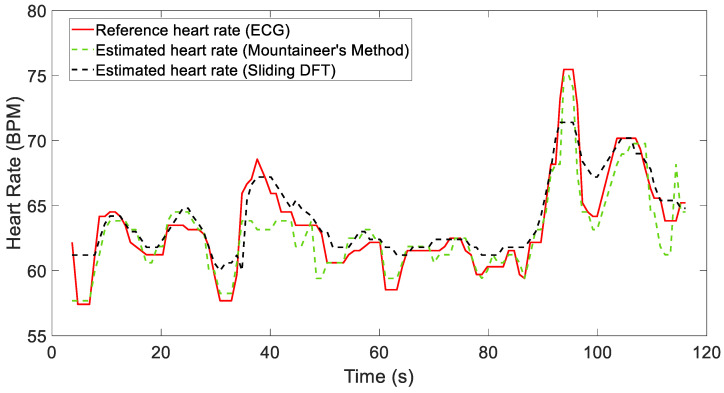
Comparison between the reference heart rate (obtained from a simultaneous ECG) and the estimation results for Recording 5.

The results demonstrate how both algorithms satisfactorily estimate the heart rate while reducing motion artifacts, with average absolute errors of 1.586 BPM and 1.749 BPM for the MM and SDFT, respectively. Figure 7 shows the estimated HR traces of both algorithms for Recording 5. To eliminate any residual false detection, a fifth-order median filter was also applied to the signals.

### 5.2. Results Using the Driving Simulator

We carried out an experiment in our BioSensLab laboratory involving a total of twelve subjects (2 women and 10 men), who were students at the University of Udine. Their ages were in the [21,34] range. We asked them to read and sign an informed consent to log their physiological signals. The sensors were positioned on the neck for the PPG recordings and on the chest for the ECG recordings (see Figure 2). The driving simulator employed in our experiment consists of a three-axis moving platform (DOF Reality Professional P3) with a curved screen, a virtual reality (VR) (Oculus Rift, Meta, Menlo Park, CA, USA) headset, a racing seat, and a force-feedback steering wheel complete with pedals and a gearbox (Logitech G29, Lausanne, Switzerland & San Jose, CA, USA). This setup enabled a highly immersive driving experience for the individuals. For the driving simulator software, we used VI-DriveSim [32] from VI-grade (Tavagnacco, Udine, Italy).

**Figure 8:**
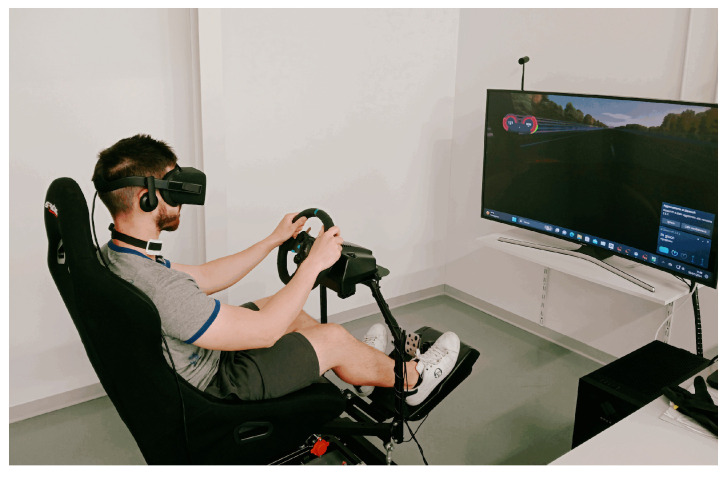
A participant in the driving test scenario using our simulator.

First of all, we asked the subjects to get in the simulator and drive a car with an automatic transmission on a 14 km highway along which we added six obstacles that the subjects had to go through while driving (see Figure 8). In particular, the complete track had six road sections characterized by roadworks indicated by Jersey barriers. The length of each obstacle was 200 m, and they were spaced 2 km from each other. The first obstacle was also located 2 km from the beginning of the course. We also asked the subjects to drive as they would normally do in a real-world scenario. In this way, they completed this experiment phase in about 7 min, with an average speed of about 120 km/h. After this phase was concluded, the subjects were asked to get out of the simulator.

With the aim of monitoring the health and well-being of drivers, the acquired signals were used to estimate the heart rates of drivers using both algorithms. As can be seen from Table 2, both algorithms perform well, even in more dynamic situations compared to simply sitting at a desk and moving one’s head. They satisfactorily attenuate motion artifacts and accurately estimate the heart rate, which in this scenario experiences greater variations than in the previous experiment.

**Figure 9:**
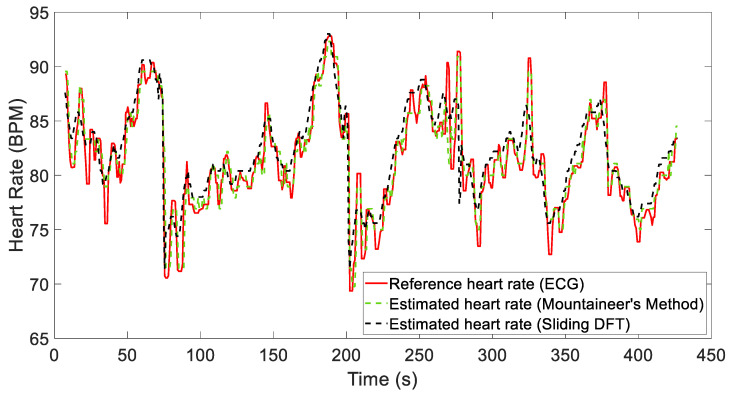
Comparison of reference heart rate (obtained from simultaneous ECG) and estimation results for Recording 12.

**Figure 10:**
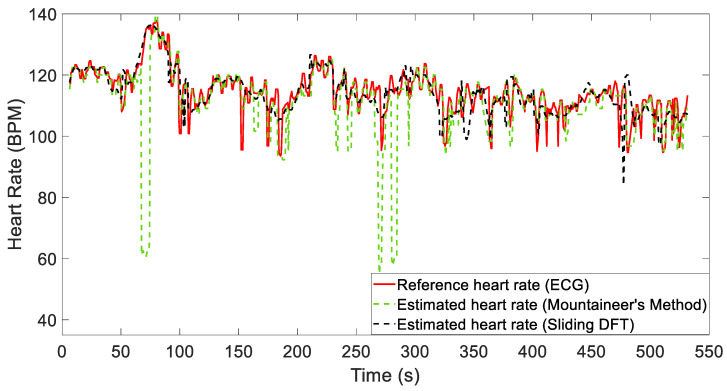
Comparison of reference heart rate (obtained from simultaneous ECG) and estimation results for Recording 2.

Figure 9 and Figure 10 show the results obtained considering two different recordings, i.e., Recordings 12 and 2. These figures compare the heart rate estimates from both algorithms with the reference values extracted from the ECG signal.

For Recording 12 in particular, both algorithms reliably estimate the heart rate, as demonstrated by comparing it with the reference signal (see Figure 9). The metrics reported in Table 2 show only minor discrepancies, thus indicating good performance. However, as shown in Figure 10, significant local artifacts may occur, leading to more substantial estimation errors. These situations might require a strategy that considers the long-term behavior of the signal. In the future, we plan to use both algorithms in a cascading manner to refine this estimation, enabling one to validate the other. By doing so, we will obtain two simultaneous estimates for comparison. If a significant discrepancy persists, we will be able to identify any malfunctions. Despite these challenges, the results remain acceptable, and the heart rate is generally estimated with sufficient accuracy. A fifth-order median filter was applied to these signals for representation.

### 5.3. Results Using a Public Dataset

To ensure the reliability, validity, and applicability of the two implemented algorithms in different contexts, we also tested them on the dataset from [20]. We chose this dataset, even though many others are available online for different types of activities [21,22], because it is currently the one that best aligns with our goal.

However, it is important to highlight that our data differ significantly from those collected in the considered dataset and, in general, from those available in the literature, which are gathered from wrist-worn PPG sensors used in physical activity settings. They are, in fact, highly sensitive to dynamic movements, which makes them prone to motion artifacts, potentially resulting in less reliable data.

In particular, the considered public dataset contains single-channel PPG signals and ECG signals from 12 male subjects with yellow skin and aged between 18 and 35 years. For each subject, the PPG signal was recorded from the wrist using a pulse oximeter with a green LED. The ECG signal was recorded from the chest using wet ECG sensors. During data recording, subjects walked or ran on a treadmill at the following speeds: speed of 1–2 km/h for 0.5 min, speed of 6–8 km/h for 1 min, speed of 12–15 km/h for 1 min, speed of 6–8 km/h for 1 min, speed of 12–15 km/h for 1 min, and speed of 1–2 km/h for 0.5 min.

**Figure 11:**
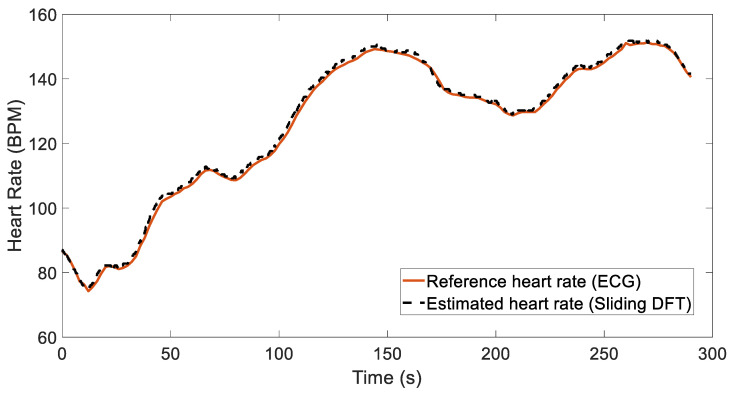
Comparison of reference heart rate (obtained from simultaneous ECG) and estimation results for Recording 9.

As mentioned before, the dataset from [20] and the others available online involve more dynamic movements, and for these data, the mountaineer’s method seems to have issues, resulting in higher errors compared to the sliding DFT. In fact, the mountaineer’s method was specifically implemented in the microprocessor with the aim of estimating the HRs from data collected by the necklace sensor worn by drivers, which were subjected to minimal motion artifacts. On the other hand, the SDFT works acceptably, as can be seen from Table 3. This table presents the values of the three metrics that were already described for the other two datasets, summarizing the performance of the recordings available in the public dataset. The only difference in the implementation of the SDFT between the data acquired with the necklace sensor and the data available online lies in the setting of the $\Delta_{max}$ parameter. This parameter defines the maximum variation in BPM between consecutive samples in order to restrict the heart rate search range. In particular, in the former case, $\Delta_{max}$ was set to 10 BPM for each overlapping window. However, for the dataset in [20], it was necessary to adjust this parameter for 3 out of the 12 individuals (Recordings 2, 6, and 10) based on their characteristics, resulting in us choosing values of 3, 21, and 8 BPM, respectively. Finally, Figure 11 shows the results obtained for Recording 9 by applying the sliding DFT (we chose it because it provides us with high-performance results and has often been considered in other studies in the literature). Here, we compare the heart rate estimate with the reference value extracted from the ECG signal.

## 6. Discussion

Our research focuses specifically on using the developed necklace PPG sensor to collect data from drivers, who typically exhibit limited movements. These data are quite different from those collected from wrist-worn PPG sensors employed in situations involving physical activity. Our necklace sensor has a more stable interaction with the skin surface and is less affected by movement, as it rests against a relatively stationary area of the body. This placement is ideal for acquiring data in a controlled environment such as a car, where the driver’s movements are minimal. Wrist-worn PPG sensors, instead, are more sensitive to dynamic movements and external factors due to the constant movement of the wrist during physical activity. This can introduce significant motion artifacts, leading to less reliable data.

From the experimental results obtained using our necklace sensor (see both Section 5.1 and Section 5.2), we can observe that both algorithms estimate heart rate satisfactorily. This is confirmed by the estimation error metrics calculated for each recording, which show significantly low values.

Hence, our necklace sensor data benefit from a relatively stable environment with fewer motion artifacts. This stability enhances the accuracy of heart rate measurements and ensures high-quality PPG signals with minimal interference. Regarding the considered public dataset, it was collected using a wrist-worn PPG sensor during physical activities, thus involving a challenging context. Individuals often engage in rapid and multidirectional movements, causing substantial motion artifacts that can interfere with the accuracy of the PPG signal. Indeed, wrist-worn PPG sensors are versatile but struggle to maintain signal integrity during intense activities. This can lead to inaccuracies in the monitored physiological parameters. For this reason, as reported in Section 5.3, the mountaineer’s method appears to experience issues, leading to higher errors when compared to the sliding DFT. On the other hand, the SDFT works acceptably, as can be seen from the metrics reported in Table 3. Note that, however, the performance of the procedure described in [4] is higher than the one obtained in our implementation. This is due to the fact that in [4], a more elaborate system is employed to estimate HR that takes into account signals recorded by an accelerometer and processed using a Wiener filter and other steps. Our aim, however, is to propose a sensor using a low-power microprocessor that allows for acceptable estimation of the heart rate. To further refine this estimation, in the future, we plan to implement both algorithms in a cascading fashion, allowing one to validate the other. This would be feasible given a low computational cost. Therefore, it would enable us to have two simultaneous estimates for comparison. If a significant discrepancy between the two estimates persists over an extended period, we would be able to identify that something is not working correctly and take appropriate countermeasures.

## 7. Conclusions

This paper introduces the design of a novel PPG signal acquisition system operating in the R and IR bands. The focus is on continuous monitoring of the HR, i.e., a vital signal for assessing drivers’ well-being. The sensor is worn as a necklace and captures signals from the driver’s neck, minimizing interference with driving movements while ensuring high-quality signal acquisition. It provides a significant advantage over wrist-worn PPG sensors used in physical activity settings, which often face challenges due to dynamic movement and increased motion artifacts. This distinction underscores the importance of sensor placement and the environment in which data are collected and ultimately leads to more reliable and application-specific insights. Lab tests were conducted using a driving simulator, demonstrating the system’s effectiveness and robustness against motion-related artifacts. To test the sensor’s performance, two low-computational-cost algorithms for HR estimation were implemented and integrated into it: the mountaineer’s method and the sliding DFT, which are characterized by extremely reduced computational times. In the future, they will allow us to estimate the heart rate in parallel, enabling one to validate the other. Moreover, the system emphasizes a low-power design and a streamlined algorithm. Both algorithms provided heart rate estimates that were quite accurate compared to the reference signals recorded by an ECG sensor. They were tested on a public dataset as well, which contained data similar to those obtained during our tests with our necklace sensor. The results show that while the sliding DFT continues to exhibit satisfactory performance, despite requiring some adjustments in a few recordings related to the search range parameter for the heartbeats, the mountaineer’s method encounters problems. These occur because the signals from the public dataset are more susceptible to noise and motion artifacts since they are recorded using a wrist sensor. Future work will also consider gathering data during real-world driving, including in complex urban scenarios and other situations.

## Figures and Tables

**Table 1:** Average absolute error, standard deviation of the absolute error, and average relative error in BPM considering all 10 recordings of the subject tested during our preliminary tests for both the MM and SDFT algorithms.

	*av*AE		*sd*AE		*av*RE	
**Rec**	**MM**	**SDFT**	**MM**	**SDFT**	**MM**	**SDFT**
1	1.865	2.616	2.718	2.820	0.028	0.039
2	2.043	2.179	2.420	2.424	0.031	0.033
3	0.909	1.589	0.843	1.083	0.014	0.025
4	1.265	1.542	1.464	1.490	0.02	0.024
5	1.097	1.169	1.162	1.164	0.017	0.018
6	1.197	1.088	1.880	1.883	0.019	0.017
7	0.732	1.315	0.663	1.258	0.012	0.021
8	1.183	1.422	1.914	1.929	0.018	0.022
9	3.582	2.926	4.896	5.168	0.053	0.030
10	1.985	1.640	3.790	3.806	0.030	0.026
Mean	1.586	1.749	2.175	2.265	0.024	0.026

**Table 2:** Average absolute error, standard deviation of the absolute error, and average relative error in BPM considering all 12 subjects who drove the driving simulator for both the MM and SDFT algorithms.

	*av*AE		*sd*AE		*av*RE	
**Rec**	**MM**	**SDFT**	**MM**	**SDFT**	**MM**	**SDFT**
1	2.214	2.305	2.184	2.186	0.024	0.026
2	4.093	3.699	9.574	9.582	0.036	0.033
3	2.033	1.799	6.080	6.084	0.021	0.017
4	1.935	2.613	2.212	2.314	0.028	0.044
5	1.095	1.401	1.228	1.266	0.018	0.024
6	1.203	1.543	0.994	1.050	0.009	0.013
7	1.643	2.834	2.805	3.048	0.025	0.043
8	4.218	2.985	8.052	8.146	0.061	0.043
9	1.611	2.152	2.097	2.166	0.021	0.028
10	1.451	2.508	1.477	1.816	0.014	0.024
11	0.920	1.131	2.011	2.022	0.010	0.014
12	0.889	1.670	1.070	1.324	0.011	0.021
Mean	1.942	2.220	3.315	3.417	0.023	0.026

**Table 3:** Performance metrics for the group of recordings for sliding DFT and the reference paper [4].

	SDFT			Ref Paper [4]	
**Rec**	***av*** **AE**	***sd*** **AE**	***av*** **RE**	***av*** **AE**	***sd*** **AE**
1–12	1.86	2.36	0.02	0.65	1.00

## Data Availability

The data presented in this study are available on request from the corresponding author.

## Abbreviations

The following abbreviations are used in this manuscript:

HR	Heart Rate
HRV	Heart Rate Variability
ECG	Electrocardiogram
PPG	Photoplethysmography
BPM	Beats Per Minute
MM	Mountaineer’s Method
SDFT	Sliding Discrete Fourier Transform
PCB	Printed Circuit Board
PTT	Pulse Transit Time
LSB	Least Significant Bit
